# Findings From the National Machine Guarding Program–A Small Business Intervention: Machine Safety

**DOI:** 10.1097/JOM.0000000000000836

**Published:** 2016-09-09

**Authors:** David L. Parker, Samuel C. Yamin, Min Xi, Lisa M. Brosseau, Robert Gordon, Ivan G. Most, Rodney Stanley

**Affiliations:** HealthPartners Institute, Bloomington, Minnesota (Dr Parker, Mr Yamin, Dr Xi, Mr Gordon); University of Illinois at Chicago, School of Public Health, Division of Environmental and Occupational Health Sciences (Dr Brosseau); University of New England, School of Public Health (Dr Most); and MEMIC, Loss Control Division, Portland, Maine (Mr Stanley).

## Abstract

**Objectives:**

The purpose of this nationwide intervention was to improve machine safety in small metal fabrication businesses (3 to 150 employees). The failure to implement machine safety programs related to guarding and lockout/tagout (LOTO) are frequent causes of Occupational Safety and Health Administration (OSHA) citations and may result in serious traumatic injury.

**Methods:**

Insurance safety consultants conducted a standardized evaluation of machine guarding, safety programs, and LOTO. Businesses received a baseline evaluation, two intervention visits, and a 12-month follow-up evaluation.

**Results:**

The intervention was completed by 160 businesses. Adding a safety committee was associated with a 10% point increase in business-level machine scores (*P* < 0.0001) and a 33% point increase in LOTO program scores (*P* < 0.0001).

**Conclusions:**

Insurance safety consultants proved effective at disseminating a machine safety and LOTO intervention via management-employee safety committees.

## Abstract 2

Supplemental Digital Content is available in the text

The Occupational Safety and Health Administration (OSHA) has several standards related to machine safety. These include requirements for machine guarding,^1^ lockout/tagout (LOTO),^2^ control of mechanical power transmission hazards,^3^ and for specific machines such as power presses^4^ and abrasive wheels.^5^ The absence or incomplete use of machine guarding or failure to implement a LOTO program may result in serious traumatic injuries including amputations and fatalities.^6–9^ LOTO consistently ranks as one of the most frequently cited OSHA standards in manufacturing (NAICS 31, 32, 33).^10^ Citations are also common for violations of the OSHA machine guarding standard and other machine-related regulations.^10^

From 2002 to 2007, we conducted the Minnesota Machine Guarding Study (MN-MGS), an intervention effectiveness trial in 40 small (5 to 100 employees) metal fabrication firms in the Minneapolis-St. Paul metropolitan region. At baseline, machines frequently lacked point of operation and other critical safeguards.^11,12^ Participants received on-site training and a report with detailed recommendations for improving machine guarding and related programs such as LOTO. One-year follow-up assessments found improvements of 7.5% points (from 58.5% to 66.0% in the half of the businesses with lowest baseline scores) in machine guarding and 12.0% points (52.3% to 64.3%) in safety programs.^11^

The National Machine Guarding Program (NMGP) is a translation research intervention designed to convert findings from the MN-MGS into prevention programs that can be readily implemented by small businesses. In this paper, we describe positive changes in machine guarding and machine safety programs for the 160 businesses that completed the intervention.

## METHODS

An insurance safety consultant obtained informed consent from each business owner before enrollment. The institutional review boards of the Park Nicollet Institute and University of Illinois at Chicago approved all study methods and materials. Participation did not entail exemptions from OSHA enforcement-related activities and no monetary incentives were given including discounts for workers’ compensation premiums.

The NMGP was developed and implemented in partnership with two workers’ compensation insurance companies. We then conducted multiple two-day in-person trainings with insurance safety consultants. Training included the assessment of machine-related hazards such as point of operation guarding, the identification of unguarded moving parts, job hazard analysis (JHA), and LOTO as well as a review of study protocols. Training took place at technical colleges where there were opportunities for assessment of metal fabrication equipment.^13,14^

Safety consultants were responsible for business recruitment, evaluation, and intervention delivery. The intervention was carried out between January 2012 and September 2014. Participating businesses were recruited from among each insurer's workers’ compensation clients. Eligible businesses had metal fabrication as their primary (≥75%) source of revenue and 3 to 150 employees at the participating site. Each safety consultant was asked to identify and solicit the participation of businesses willing to participate in the study. The final sample reflected the geographic distribution of businesses receiving workers’ compensation insurance from the two insurers.^13^

### Machine Safety Audit

At baseline and follow-up, consultants randomly selected a unique sample of 12 machines for a standardized assessment of machine safeguarding. Evaluation was performed at each of the selected machine workstations using technical checklists developed and tested in the MN-MGS.^15^ Checklists included yes/no questions within four categories: equipment safeguards, LOTO procedures, electrical hazards, and work environment. Checklists varied by machine type and contained between 25 and 35 questions each depending on the complexity of the machine. Sample checklists are found online in “Appendix 1”.

### Safety Management Audit

At baseline and follow-up, a safety management audit checklist was completed during an interview with the owner or the owner's representative. The safety management audit addressed four areas: safety leadership, JHA, machine maintenance, and LOTO (“Appendix 2,”).

As part of the audit, documentation was reviewed for written safety programs and policies. For all checklist items, a “yes” response meant that the evaluator verified the presence of a safeguard, policy, or written document. In addition, during the safety management audit, demographic data were collected about each business, including zip code, years in business, number of employees, and the owner's education.

### Intervention Activities

Participating businesses received four visits from an insurance safety consultant: a baseline safety audit, intervention visits at three and six months postbaseline, and a follow-up audit at 12 months. At the conclusion of the baseline evaluation, data were entered into the software developed for the study, and the owner and/or safety committee received a summary report of findings.

The safety consultant and owner used results from the summary report to develop a one-year business action plan. Owners selected specific areas to address in conjunction with guidance from the safety consultant. If an employee-management safety committee was not present, owners were encouraged to create one as an initial step. Other recommendations included improving machine guarding, LOTO, and conducting job hazard analyses. For items selected as part of the action plan, the owner assigned responsibility to one or more employees and a target date was set for completion. Guidance materials, such as written policy templates, were provided to assist businesses with implementing recommendations for safety leadership, machine guarding inspections, LOTO, and JHAs.

The three and six-month visits consisted of encouraging the owner or safety committee to continue to complete their business action plan and providing supporting materials if needed. At the completion of the three and six-month site visits, safety consultants electronically entered data on recommendations and progress for each shop into an intervention activity recordkeeping sheet within the software. In some instances, either the three- or six-month visit, but not both, was conducted via telephone. The telephone consultation consisted of a review of the business action plan to remind the owner to continue working to meet pre-determined goals and as an opportunity for the shop to request technical guidance.

### Analysis

Audit results and intervention activity records were transmitted electronically from field sites to the research team and analyzed using SAS version 9.2 (SAS Institute Inc., Cary, NC).^16^ Power was computed using business-level machine safety scores from the MN-MGS while accounting for variance within and between shops using a linear mixed model. Our sample size of 150 provides a power of over 0.8 to detect a 5% to 10% improvement in machine safety score.

Analyses of NMGP data included mean and standard deviations for continuous variables, and frequencies and percentages for categorical variables. Bivariate analyses including Chi-square, *t* test, analysis of variance (ANOVA), and Pearson correlation coefficients were used to explore the relationship between percentage of missing items on machine safety checklists and business demographics. Multiple regression was used to explore the relationship between machine age and percentage of missing items on machine safety checklists. Regression modeling was also used to examine the relationship between different aspects of machine safety and the presence of administrative programs such as a safety committee and written policies related to safety and health.

The machine safety checklists were used to create two summary scores:

- Business-level machine score: The number of “yes” responses for all machines was divided by the number of “yes” plus “no” responses on the 12 machine safety checklists completed at each shop to compute a single score.
- Machine-level score: The number of “yes” responses was divided by the number of “yes” plus “no” responses for individual machine checklists.

In addition, four subcategory scores were calculated for each machine. Scores were calculated as the number of items present divided by the total number of items x 100:

- Equipment safeguards: Depending on the type of machine, different numbers of items were used to assess point of operation safeguards, safeguards for other mechanical hazards, power transmission guards, workpiece control, operational controls and emergency stops, and the presence of lockable disconnects.
- LOTO procedures: Five items addressed the presence and completeness of LOTO procedures.
- Electrical hazards: Six items addressed the condition and configuration of electrical wiring.
- Work environment: Between six and eight items addressed conditions of the work area and employee work practices such as wearing proper safety eyewear. Work practices were only assessed if a worker was present at the workstation at the time a machine was evaluated.

An overall safety management audit score was created using the 33 questions from the safety management audit as well as separate measures as described below. Scores were calculated as the number of items present divided by the total number of items × 100.

- Safety leadership: Twelve questions assessed the safety management structure, written safety programs, and workplace safety policies. Safety leadership was defined as a formal, organized structure within which employees and management cooperatively identify, evaluate, and remediate hazards.
- JHA: Eight questions determined the presence and completeness of a program for conducting JHAs and integration of findings from JHAs into regular work practices. JHA was defined as a systematic means of assessing hazards associated with each job and devising means of remediating the hazards
- Machine maintenance program: Eight questions assessed the documentation of periodic inspection of machines to ensure they were effectively guarded. Machine maintenance was defined as inspecting machines on a routine basis to ensure safe operation.
- LOTO: Five questions assessed key elements of a LOTO program and related employee training and record keeping. LOTO was defined as compliance with OSHA standard 1910.147 to ensure safe control of hazardous energy. OSHA requires that each business have a comprehensive written LOTO program. A LOTO procedure is a series of steps to safely shut down and restart machines.

## RESULTS

A total of 221 businesses (198 enrolled by insurer A and 23 by insurer B) received a baseline safety audit. Of these, 160 (72%; 146 from insurer A and 14 from insurer B) completed the entire program. The most common reason for leaving the study was switching to another workers’ compensation carrier (61%; 37/61). Fifteen businesses left the study citing a lack of time and/or interest. The investigators removed nine businesses because corporate restructuring by insurer A made it impossible for safety consultants to complete all intervention activities on a one-year timetable in accordance with the study protocol.

There were no significant differences between shops that completed the intervention compared with those that did not with regard to mean shop size (*P* = 0.32), business-level machine score (*P* = 0.89), or safety management audit score (*P* = 0.79). In addition, the baseline shop and baseline machine scores did not vary between shops that started during the first and second halves of the intervention period (*P* > 0.10).

For the 160 businesses that completed the intervention, baseline business characteristics did not differ between insurers A and B with regard to the business-level machine score (74% vs 71%; *P* = 0.26) or safety management audit score (42% vs 48%; *P* = 0.22). Final analysis was performed on the combined sample of 160 shops. As summarized in Table 1, participants were drawn from a wide geographic area. The majority of businesses had fewer than 30 employees (68%) and one-third (34%) had a safety committee at baseline.

Baseline measures for the overall machine score did not differ on the basis of the owners’ years of experience in metal fabrication or level of technical or general (eg, high school, college) education. Similarly, there was no difference in the overall shop score when the data were stratified by different levels of these variables.

A total of 1912 machines was evaluated at baseline and 1913 were assessed at follow-up. The average business-level machine score increased from 73% to 79% (*P* < 0.0001) over the course of the intervention. Point of operation guards increased from 67% to 72% (*P* < 0.0001) and the presence of lockable disconnects rose from 88% to 92% (*P* < 0.0001). LOTO procedures showed the largest improvement from 8% to 33% (*P* < 0.0001) (Table 2).

Year of manufacture was obtained for 837 machines at baseline and 714 at follow-up (Table 3). There was a negative trend in the level of safeguarding with increasing age (*P* trend < 0.0001). Over the course of the intervention, there were small improvements in the equipment safeguard score for all types of machines except milling/drilling/boring. The latter group was also the oldest machine type at 32 years on average (SD = 13), compared with 22 years for all machines (SD = 16). For the 66 shops in which age was known for at least six machines at baseline, there was a slight correlation between machine age and years in business (*R*^2^ = 0.08; *P* = 0.01), and no correlation between machine age and the number of employees (*R*^2^ = 0.003; *P* = 0.64) (data not shown in a table).

The overall safety management score showed a positive trend with increasing business size at baseline (*P* trend < 0.0001) and follow-up (*P* trend < 0.001) (Table 4). From baseline to follow-up, there were improvements in the overall safety management score in all business size ranges (*P* < 0.0001 for all groups). Businesses in all size ranges also made improvements in safety leadership and LOTO. Machine maintenance program scores improved significantly for all but the largest shops (*P* = 0.15). At baseline, JHAs were infrequently conducted regardless of business size. There was an improvement of 15% points in mean JHA score for all shops combined (*P* < 0.0001) and significant improvements within each business size range for all but the smallest (3 to 10 employee) shops.

At baseline, 34% of companies had a safety committee compared with 58% at follow-up (*P* < 0.0001) (data not in tables). For businesses lacking a committee at baseline (*N* = 105), larger firms were more likely than their smaller peers to add one. For businesses with 3 to 10, 11 to 29, 30 to 49, and 50 to 150 employees without a safety committee at the outset 7/36, 21/51, 9/13, and 5/5 respectively, added one (*P* for trend < 0.0001).

As summarized in Table 5, businesses that started and ended the intervention with a safety committee attained the highest scores on the overall safety management audit and its four component scores (with questions on safety committee removed from these outcome measures) at baseline and follow-up. Shops that added a safety committee made substantially greater gains in the overall safety management audit score than shops that did not (24 vs 9% point improvement; *P* = 0.0002). Differences in improvements between these two groups was borderline significant for the LOTO program (*P* = 0.06) and JHAs (*P* = 0.06) and not significant for machine maintenance programs (*P* = 0.83).

As summarized in Table 6, businesses that added a safety committee during the study also made substantially greater improvements in this measure when compared with businesses that did not have a safety committee throughout the study period (10 vs 2% point improvement; *P* = 0.0001). For the four businesses that went from having a safety committee to not having one over the course of the intervention, there were minimal, nonsignificant changes between baseline and follow-up for the in businesses-level machine, safety management audit, and safety leadership scores (*P* ≥ 0.6 for all measures).

Regression analysis was used to compare shops that started without a safety committee (*n* = 105) and ended the intervention with (*n* = 42) or without (*n* = 63) one. Controlling for baseline safety management audit score and business size, shops that added a safety committee improved 21% points more on the overall safety management audit score (*P* < 0.0001) and 9% points on the business-level machine score (*P* < 0.0001)) when compared with those that did not add a committee.

## DISCUSSION

Several authors have developed frameworks for disseminating health and safety programs to small enterprises through intermediary organizations such as insurers.^17–20^ However, there is a lack of supporting data to test suggested best practices due to the difficulties and expense entailed in implementing a large-scale standardized intervention. The success of the NMGP in improving both machine guarding and LOTO demonstrates the potential for intermediaries such as insurance safety consultants to effectively work with small businesses to effect positive safety-related changes. Insurance personnel are able to provide technical information and consultative services in an unbiased fashion and without the need to promote commercial products. In addition, they are the most common source of safety information used by small businesses (74%), followed by state OSHA consultation (38%).^21^

Owners often rely on information obtained through informal relationships with individuals who they feel they can trust.^22,23^ These individuals may be vendors with a vested interest in a specific product that is inadequate to meet the needs of employees and employers. However, it is often difficult for employers or employees to assess the quality of information provided, the efficacy of personal protective equipment, or whether consultative services adequately meet their needs.^17,22,24^

The lack of an infrastructure for human resource management is another important problem faced by small businesses.^25–27^ Accounting, finance, production, and marketing take precedence over personnel-related issues and personnel policies—including those that address safety—are frequently lacking in firms with fewer than 50 employees.^26,27^ Although it is commonly supposed that lack of resources is the primary barrier to safety performance in small businesses, the true picture is considerably more complex.

For example, Champoux and Brun^28^ found that a lack of resources is not likely the major obstacle to improving business health and safety in most small businesses. In the NMGP, larger businesses were more likely to have a safety committee at the start of the intervention or establish one over the course of the intervention. There was, however, no indication that the need for a safety management structure varied with business size. After controlling for the presence or absence of a safety committee, business size (range 3 to 150 employees) did not have an impact on any of our safety measures. In addition, although aging machinery was associated with lower safeguarding audit scores, there was no association between machine age and only a slight correlation with years in business.

It is apparent from the NMGS that when encouraged to establish a safety infrastructure, among smaller businesses (<150) size appears to have a little effect. Engaging owners and workers is particularly important for small businesses where owners are the gateway to shops, make decisions about the selection and purchase of controls, and set and enforce rules and policies.^28–31^ In this study, after completion of a summary audit report, safety consultants encouraged the owner and safety committee to work together in selecting areas for improvement and developing a one-year business action plan.^32,33^

The NMGP demonstrated the importance of having or adding a adding a safety committee in improving summary measures of machine safety. Businesses with a safety committee had the highest baseline and follow-up summary scores. Business that added a safety committee improved summary scores substantially more than those that started and ended without one. Regardless of size, adding a safety committee was likely to result in substantially more improvement than not doing so.

Coordinated worker and owner participation is crucial to the identification of hazards and subsequent selection and implementation of controls.^23,28,33–37^ A safety leadership structure centered on a safety committee appeared to contribute to improvements in several critical workplace safety measures.^13,14,38,39^ This entails shared responsibility between workers and management and was central to the NMGP intervention. Although there is a debate on the optimal characteristics of a safety committee, shared responsibility seems central to most.^21^ Regardless, many shops with safety committees had substantial room for improvement in most summary measures, indicating the need for other elements of safety management beyond the presence of a safety committee.

The magnitude of machine-related changes is hard to assess. Because stationary machines require some level of guarding and many require lockout procedures during repair and maintenance, even small improvements have the potential to positively affect changes. As of 2013, there were more than 83,000 metalworking establishments in the United States, employing 2.8 million workers.^40^ We estimate that these businesses were operating 8.3 million machines. Improving LOTO by 20% as seen in the NMGP has the potential to positively impact almost 1.7 million hazardous machines. Similarly, a 10% point improvement in machine guarding has the potential to substantially improve safety.

## LIMITATIONS

As a pragmatic trial, this intervention emphasized the best possible design and did not include a control group. In developing real-world intervention strategies for small manufacturing firms, study design must take into account the problems related to randomization, accessing establishments, cost, and outcome measurement, as well as the needs of owners and workers. Pragmatic trials evaluate the effectiveness of interventions in order to maximize applicability of the trial's results to routine settings.^41,42^

Addition of a control or delayed intervention group would have entailed obtaining a baseline measure from the control shop. Once baseline measures are obtained, we believe it is unethical to not provide the results to the business when the hazards are known to cause catastrophic injury. All of our previous intervention studies showed that order of recruitment was not predictive of baseline or outcome measures and time may be treated as a covariate in analysis.

It was not possible to monitor the daily interactions between safety consultants and business participants. Although electronic forms were used to formulate an action plan at the baseline visit and to track activities during subsequent intervention visits, there were no records as to when a specific problem may have been remediated or the underlying motivating factors.

Perhaps the greatest problem related to the NMGP is long-term sustainability. One of the participating insurers experienced competing demands on safety consultants’ time and changing priorities within the company and declined to continue the program. Thus, although there were clear improvements in safety outcome measures, the need for long-term institutionalization is crucial and by no means assured.^43^

## CONCLUSIONS

The NMGP highlights the need for a nationwide effort to improve many aspects of machine safety within small industrial firms. Sustainable improvements would substantially reduce risk for serious workplace trauma and work-related fatalities. The NMGP provides a framework for comprehensively auditing and improving risk management practices and demonstrates the central role of worker participation and representation.^29^ An important first step is to improve or implement worker-management safety programs.

With regard to occupational safety and health research in small-scale enterprises, there is a need to fund long-term pragmatic intervention studies. The NMGP represents an important step in translating the findings of a controlled trial (MN-MGS) and carried out in a small region to a widely applicable intervention program that can be integrated into the routine work of intermediary organizations such as insurance companies and delivered to small businesses. Future research should evaluate whether or not gains such as those achieved in the NMGP are independently sustained by small businesses with minimal ongoing assistance. Work also needs to be done on developing effective outreach programs that do not require time-intensive in-person consultative services.

## Supplementary Material

Supplemental Digital Content

## Supplementary Material

Supplemental Digital Content

## Figures and Tables

**TABLE 1:** Business Characteristics at Baseline (*N* = 160)

Geographic regions	
Northeast: CT, DE, MA, ME, NH, NJ, NY, PA, VT	35
Southeast: AL, AR, FL, GA, KY, NC, SC, TN, VA	30
North central: IA, IL, IN, MI, MN, SD, WI	74
Southwest: AZ, KS, MO, NE, NM, TX	21
All shops completing the intervention	160
Number of employees	
3–10	44
11–29	65
30–49	22
50–150	29
Mean number of employees	29
Safety committee status at baseline	
Number and percent with a safety committee	55 (34%)

**TABLE 2:** Business-Level Machine Audit Scores (*n* = 160) Based on 12 Randomly Selected Machines per Establishment: Baseline and 12-Month Follow-Up

	Baseline		12-Month Follow-Up		Change		
Evaluation Measure	Mean %	SD	Mean %	SD	Percentage Point Change	SD	*P*
Business-level machine score	73	9	79	11	6	10	<0.0001
Equipment safeguards	81	10	83	10	2	8	<0.0001
Point of operation safeguards	67	20	72	19	5	18	0.0023
Safeguards for other mechanical hazards	73	16	75	14	2	14	0.0381
Power transmission guards	92	12	94	10	2	9	0.0002
Workpiece control	83	16	84	15	1	16	0.2531
Operational controls and emergency stops	83	11	84	12	1	10	0.0585
Lockable disconnects	88	18	92	17	4	17	<0.0001
LOTO procedures	8	22	33	42	25	43	<0.0001
Electrical	92	8	95	7	3	9	<0.0001
Work environment	90	9	93	8	3	9	0.0002

SD, standard deviation.

**TABLE 3:** Machine Age and Equipment Safeguarding at Baseline and Follow-Up

	Machine Age in Years				*P* for Trend Across Age Strata	Machines Age Known	Machines Age Unknown	*P* Age Known vs Unknown
	≤10	11–25	26–49	≥50				
Equipment safeguards								
Mean % baseline	92	87	77	72	<0.0001	85	77	<0.0001
Number at baseline	238	319	228	52		837	1075	
Mean % follow-up	95	90	79	69	<0.0001	89	79	<0.0001
Number at follow-up	224	326	128	36		714	1199	
*P* for change between baseline and follow-up	0.005	0.01	0.16	0.57		<0.0001	0.0005	

**TABLE 4:** Baseline and Follow-Up Safety Management Audit Scores Stratified by Business Size

			Safety Leadership		JHA^*^		Machine Maintenance		LOTO^†^		Overall Safety Management Score	
Number of Employees	*N*	Intervention Status	Mean %	SD	Mean %	SD	Mean %	SD	Mean %	SD	Mean %	SD
All shops	160	Baseline	58	23	10	25	43	28	55	37	43	21
		Follow-up	73	26	25	39	58	29	76	33	59	24
Percentage point increase			15	21	15	39	15	30	21	37	16	19
*P* for change within group			<0.0001		<0.0001		<0.0001		<0.0001		<0.0001	
3–10 employees	44	Baseline	47	26	7	24	37	30	40	40	35	25
		Follow-up	58	28	14	31	48	32	61	42	46	26
Percentage point increase			11	18	7	30	11	27	21	41	11	16
*P*			0.0001		0.1113		0.0111		0.0011		0.0001	
11–29 employees	65	Baseline	58	20	7	22	44	25	56	37	42	17
		Follow-up	71	25	22	40	60	26	80	31	59	22
Percentage point increase			13	22	15	34	16	31	24	38	17	18
*P* for change within group			<0.0001		0.0006		0.0001		<0.0001		<0.0001	
30–49 employees	22	Baseline	64	22	20	34	50	32	65	33	50	21
Post		Follow-up	85	17	47	42	76	28	90	18	74	19
Percentage point increase			21	25	27	49	26	31	25	37	24	23
*P* for change within group			0.0007		0.0132		0.0008		0.0044		0.0001	
50–150 employees	29	Baseline	71	21	12	26	45	27	66	30	51	17
		Follow-up	90	11	31	41	53	26	80	24	67	15
Percentage point increase			19	20	19	47	9	32	14	26	16	18
*P* for change within group			<0.0001		0.0263		0.1487		0.0083		<0.0001	
*P* value for trend in scores: baseline			<0.0001		0.18		0.16		0.001		0.001	
*P* value for trend in scores: follow-up			<0.001		0.01		0.12		0.005		<0.0001	

^*^Job hazard analysis.

^†^Lockout/tagout.

**TABLE 5:** Baseline and Follow-Up Safety Management Scores for Shops That Maintained (*n* = 51), Established (*n* = 42), or Did Not Establish a Safety Committee (*n* = 63)^∗^

	Baseline		Follow-Up				
Safety Committee Status at Baseline and Follow-Up	Mean %	SD	Mean %	SD	*P* for Change in Mean Scores: Baseline to Follow-Up	Percentage Point Change: Baseline to Follow-Up (SD)	*P*:^‡^ Comparison of Baseline and Follow-Up: Groups B and C
Overall safety management audit^†^							
Maintained (A)	55	19	74	15	<0.0001		
Established (B)	44	19	68	19	<0.0001	24 (21)	0.0002
Did not establish (C)	33	18	42	20	<0.0001	9 (14)	
Safety leadership^†^							
Maintained (A)	78	16	91	10	<0.0001		
Established (B)	58	20	87	14	<0.0001	29 (21)	<0.0001
Did not establish (C)	48	21	53	21	0.0915	5 (21)	
Job hazard analyses (JHAs)							
Maintained (A)	15	30	41	44	<0.0001		
Established (B)	11	27	30	41	0.0096	19 (47)	0.06
Did not establish (C)	4	18	8	26	0.2461	4 (27)	
Machine maintenance							
Maintained (A)	49	30	69	23	0.0003		
Established (B)	51	28	64	30	0.0032	13 (32)	0.83
Did not establish (C)	33	23	45	28	0.0002	12 (23)	
LOTO							
Maintained (A)	72	28	89	18	<0.0001		
Established (B)	54	39	87	26	<0.0001	33 (39)	0.06
Did not establish (C)	41	38	59	39	0.0006	18 (41)	

^*^Excludes 4 shops that went from having to not having a safety committee.

^†^Excludes checklist items concerning the presence of a safety committee.

^‡^Comparison of groups “no to yes” versus “no to no.”

**TABLE 6:** Safety Committee Status and Business-Level Machine Score^∗^

		Business-Level Machine Score						
		Baseline		Follow-Up				
Safety Committee Status at Baseline and Follow-Up	*N*	Mean %	SD	Mean %	SD	*P* for Difference in Mean Score: Baseline to Follow-Up	Baseline to Follow-Up: Percentage Point Change (SD)	*P*^†^
Yes to yes	51	75	9	81	10	<0.0001		
No to yes	42	74	7	84	11	<0.0001	10 (9)	0.0001
No to no	63	72	10	75	11	0.0367	3 (9)	
All shops completing the intervention	160	74	9	79	11	<0.0001		

^*^Number of shops “yes to no” is 4; data are not shown as a separate row in table.

^†^Comparison of groups “no to yes” versus “no to no.”
