# Supplementary material for: Findings From the National Machine Guarding Program–A Small Business Intervention: Machine Safety
Source: J Occup Environ Med. 2016 Sep 9;58(9):885–91. doi: 10.1097/JOM.0000000000000836 (PMC5010275; doi:10.1097/JOM.0000000000000836)
Supplement: Supplemental Digital Content [file joem-58-885-s001.doc]

**Appendix 1. Drill Press**

**Machine Safety Checklist**

| Machine Tag #: | Manufacturer: | Year of Manufacture: |
| --- | --- | --- |

|  | **YES** | **NO** | **N/A** |
| --- | --- | --- | --- |
| **I. Equipment safeguards** |  |  |  |
| ***Point of operation safeguards*** |  |  |  |
| Are shields in place at each point of operation? |  |  |  |
| Are shields free from cracks and in good condition? |  |  |  |
| ***Safeguards for other mechanical hazards*** |  |  |  |
| Are spring-loaded chuck keys provided? |  |  |  |
| Is the chuck guarded? |  |  |  |
| ***Power transmission guards*** |  |  |  |
| Are all moving parts below 7 ft. guarded? |  |  |  |
| Is guard free from cracks and in good condition? |  |  |  |
| ***Workpiece control*** |  |  |  |
| Are clamps provided for preventing workpiece movement? |  |  |  |
| ***Operational controls and emergency stops*** |  |  |  |
| Are all controls legibly marked? |  |  |  |
| Are all controls accessible without reaching over rotating/dangerous parts? |  |  |  |
| Are safeguards in place to prevent unintended activation of any controls? |  |  |  |
| Are all foot controls guarded to prevent unintended activation? |  |  |  |
| Are all foot control guards free from cracks and in good condition? |  |  |  |
| Is there a red mushroom-shaped emergency stop button that stops all hazardous motion? |  |  |  |
| Is an emergency stop readily accessible to each operator? |  |  |  |
| ***Lockable disconnects*** |  |  |  |
| Is a lockable disconnect in place for each energy source? |  |  |  |
| Are disconnects in plain view? |  |  |  |
| **II. LOTO procedures** |  |  |  |
| Are LOTO procedures posted on or near the machine? |  |  |  |
| *If posted, answer next 4 questions. If not, enter “no” for all 4.* |  |  |  |
| Does the LOTO procedure contain specific steps for shutting down and locking out each source of hazardous energy? |  |  |  |
| Does the LOTO procedure require that stored energy be eliminated prior to placement of lockout devices? |  |  |  |
| Does the LOTO procedure contain specific instructions for verifying the effectiveness of lockout devices and other energy control measures before maintenance is performed? |  |  |  |
| Does the LOTO procedure contain specific steps for removing LOTO devices and restoring power? |  |  |  |
| **III. Electrical** |  |  |  |
| Are all live electrical components properly enclosed and insulated? |  |  |  |
| Are all wires in good condition? |  |  |  |
| Is machine powered without the use of extension cords? |  |  |  |
| Is strain relief securely in place at both ends of drop cords? (Select “N/A” if there is no drop cord.) |  |  |  |
| Are drop cord receptacles free of knockouts, holes, or conductive materials? |  |  |  |
| Is auxiliary lighting below 7 ft. properly protected against impact? |  |  |  |
| **IV. Work practices and environment** |  |  |  |
| Is the work area free of trip hazards? |  |  |  |
| Is the machine adequately stabilized? |  |  |  |
| Is machine operator wearing safety glasses with side shields? |  |  |  |
| Are all safeguards in place when work is performed (e.g., employees do not attempt to bypass guards)? |  |  |  |
| Is machine operator's attire free of entanglement hazards? |  |  |  |
| **Notes** |  |  |  |
| Is there additional information that you believe would be helpful, to supplement your answers to the questions in this checklist? |  |  |  |
| Please use this space to describe any hazards not covered on this checklist, or to provide additional detail on any of the items in this checklist: |  |  |  |
