# Supplementary material for: Findings From the National Machine Guarding Program–A Small Business Intervention: Machine Safety
Source: J Occup Environ Med. 2016 Sep 9;58(9):885–91. doi: 10.1097/JOM.0000000000000836 (PMC5010275; doi:10.1097/JOM.0000000000000836)
Supplement: Supplemental Digital Content [file joem-58-885-s002.doc]

**Appendix 2: Safety Management Audit**

| **Safety leadership program** | **YES** | **NO** | **N/A** |
| --- | --- | --- | --- |
| Is one employee or manager designated as responsible for safety issues? |  |  |  |
| Is there a committee that discusses safety? *If "yes", answer the next 2 questions*: |  |  |  |
| If present, does this committee meet at least quarterly? |  |  |  |
| If present, are minutes from committee meetings posted? (Verify by seeing documentation.) |  |  |  |
| Are safety issues discussed at least quarterly at meetings that include all employees? |  |  |  |
| Is there a formal method for obtaining employee input on safety? |  |  |  |
| Is there a written policy stating the consequences of failing to follow safety procedures? (Verify.) |  |  |  |
| Is there a designated employee to whom injuries are reported? |  |  |  |
| Is there a written policy requiring that employees promptly report all injuries? |  |  |  |
| Is a written investigation conducted for each injury? |  |  |  |
| Is a written investigation conducted for near-misses? |  |  |  |
| Is there a policy requiring that everyone wear safety eyewear in the shop? |  |  |  |
| **Job hazard analysis program** |  |  |  |
|  |  |  |  |
| Is a program in place for conducting JHAs? *If "yes", answer the next 7 questions:* |  |  |  |
| Is there a written record of each JHA? (Verify.) |  |  |  |
| Is each job broken into successive steps or activities? |  |  |  |
| Are hazards and safe work practices/controls identified for each step? |  |  |  |
| Are ergonomic considerations (e.g., lifting and materials handling) included in each JHA? |  |  |  |
| Are JHAs reviewed and updated at least annually? |  |  |  |
| Are results of JHAs used in training employees before assigning new job tasks? |  |  |  |
| Are employees disciplined for failing to follow the control measures identified in the JHAs? |  |  |  |
| **Machine maintenance program** |  |  |  |
| Are machine safety audits conducted at least annually? (Verify by seeing documentation.) |  |  |  |
| Are machine maintenance inspections conducted at least every 60 days? |  |  |  |
| Is power outage (anti-restart) protection in place for each machine? |  |  |  |
| Are records kept showing that emergency stops are regularly inspected and tested? (Verify.) |  |  |  |
| Is there a brief audit checklist for setup of each machine? (Verify.) |  |  |  |
| Does the shop have written machine guarding policies/procedures? (Verify.) |  |  |  |
| Are light curtains or other presence-sensing devices used anywhere in the shop for machine safeguarding? *If “yes”, answer the next question:* |  |  |  |
| Is there documentation that blanking is compliant with OSHA table 0-10, or that stop-time analyses are performed, or both? (Verify.) |  |  |  |
| **Lockout/tagout (LOTO) program** |  |  |  |
| Does the shop have a written LOTO program? (Verify.) |  |  |  |
| Does the LOTO program designate "authorized" employees? (Verify.) |  |  |  |
| For each lock issued to an "authorized" employee, is there just one key that opens that lock? (Verify.) |  |  |  |
| Are there records verifying that all employees are trained in LOTO? (Verify.) |  |  |  |
| Are there records of annual audits verifying the effectiveness of written LOTO procedures for each machine? (Verify.) |  |  |  |
